# Development of Reduced‐Fat and Cholesterol‐Free Mayonnaise by Chia Seed Mucilage and Whey Protein: Quality Parameters and Organoleptic Attributes

**DOI:** 10.1002/fsn3.72064

**Published:** 2026-06-29

**Authors:** Abdollah Yousefi Sabet, Ashkan Jebelli Javan, Masoumeh Moslemi, Ali Mahdavi

**Affiliations:** ^1^ Department of Food Hygiene, Faculty of Veterinary Medicine Semnan University Semnan Iran; ^2^ Halal Research Center of IRI., Iran Food and Drug Administration Ministry of Health and Medical Education Tehran Iran

**Keywords:** chia seed mucilage, fat reduction, functional food, mayonnaise, whey protein

## Abstract

Mayonnaise is a popular condiment, but its consumption is concerning, especially for people suffering from high blood lipid levels and overweight. In this study, we aimed to formulate reduced‐fat and cholesterol‐free (without egg) mayonnaise samples with chia seed mucilage (0.5%–1%) and whey protein (5%–10%) similar to full‐fat mayonnaise in organoleptic and technical properties. Interestingly, the light mayonnaise samples showed higher emulsion stability (98.91%–99.08% vs. 57.42%), viscosity (8185–9657 vs. 8610 cp), consistency (0.73 vs. 0.65), firmness (147.8–198.2 vs. 110), and adhesiveness (1991–2170 vs. 1850 mJ), with more than a 22% calorie reduction compared to the control. The higher viscosity, along with the increased moisture content (27.95%–29.56% vs. 15.21%), resulted in a better sensory score by the panelists (4.45–4.55 vs. 3.85), likely due to the perceived creaminess and freshness. The better rheology attributes were related to the electrostatic interactions in protein‐polysaccharide complex which provide similar to or better texture than the control. The new formula showed lower brightness (due to higher light scattering), yellowness (due to lower oil content), and redness (due to egg yolk removal) compared to the control in the Hunter Lab assay. Interestingly, a lower peroxide value (3.3–3.74 vs. 5.86 meq O_2_/kg) was observed in the egg‐free and reduced‐fat samples. Furthermore, slightly more fungal growth was seen in the new formula due to its higher moisture content. However, the fungal growth remained below the maximum permitted level of 10^2^ CFU/g until the end of storage. In conclusion, the new formula containing 5% whey protein and 0.5%–1% chia mucilage can be used as light mayonnaise with appropriate technical and health promotion properties.

## Graphical abstract

The graphical abstract illustrates the development of a low‐fat, cholesterol‐free mayonnaise formulated using chia seed mucilage and whey protein as functional ingredients. Chia seed mucilage acts as a natural hydrocolloid, enhancing viscosity, water‐holding capacity, and emulsion stability, while whey protein contributes to emulsification and structural integrity. These ingredients are combined with soybean oil, vinegar, sugar, salt, and mustard powder to produce a stable oil‐in‐water emulsion through high‐speed homogenization. The synergistic interaction between chia mucilage and whey protein promotes the formation of a strong interfacial network around oil droplets, resulting in improved texture, creaminess, and product stability. The final formulation provides a reduced‐fat alternative to conventional mayonnaise while maintaining desirable sensory and physicochemical properties. The graphical abstract highlights the role of natural functional ingredients in producing a healthier mayonnaise with enhanced stability, acceptable mouthfeel, and clean‐label characteristics.

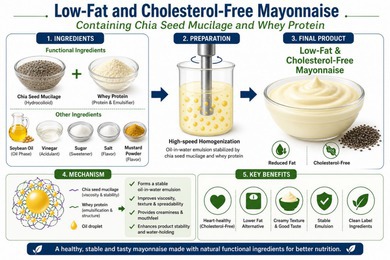

## 1 Introduction

Mayonnaise is a popular condiment worldwide, and its high consumption makes it significant in food science and nutrition. It is an oil‐in‐water emulsion containing more than 70% fat, providing high calories to consumers. From a health perspective, the cholesterol content (up to 1200 mg per 100 g of egg yolk) is a concern for healthcare systems, leading to numerous attempts to reduce the fat and specifically cholesterol levels in mayonnaise (Prabsangob and Udomrati 2024). Egg yolk contains various phospholipids that provide an appropriate emulsion. Therefore, the development of a homogeneous emulsion in the product is a concern industrially when substituting egg yolk with other alternatives. In this regard, the use of starchy materials, plant‐based proteins, and various hydrocolloids has been introduced (Bhattacharya 2023). Besides fat substitution, the addition of functional alternatives carrying bioactive compounds is of interest. For example, some edible gums are sources of antioxidants that promote health in addition to texturizing.

Chia is a plant belonging to the family Lamiaceae. A warm climate, heavy rainfall, and a mesophilic temperature between 15°C and 30°C are ideal conditions for its growth. Chia seeds offer several health benefits, including improving blood lipid profiles, lowering blood pressure and glycemic index, and providing antioxidant, antimicrobial, and immunostimulatory activity. They are a source of dietary fiber (more than 30 g per 100 g), meeting the daily requirement of 25–35 g/day for adults. Chia seeds have been introduced as egg and fat substitutes in food products due to their hydrophilic character. Chia seed mucilage is prepared by soaking the seeds in water. Its addition to foods aids in calorie reduction. The mucoadhesive property of the mucilage is utilized in developing pharmaceutical and cosmetic products (Khalid et al. 2023; Agarwal et al. 2023).

The emulsifying activity of chia mucilage is linked to proteins and fibers as hydrophilic agents and lipids as hydrophobic compounds. The proteins directly interact with water, and water molecules are entrapped within the network formed by the fibers. Due to the texturizing effect of the mucilage, it is used in various food products such as baked goods, cereal‐based foods, dairy products, and meat products. Mucilage is rich in fibers that mimic fat functionality in the matrix due to its viscosifying activity by holding the water molecules. This function provides food with softness and improved rheological behavior (Chiang et al. 2021).

Ribes et al. (2021) substituted the fat in yogurt with 2.5%, 5.0%, and 7.5% w/v chia mucilage. They found that using 7.5% w/v chia mucilage in the matrix reduced yogurt syneresis compared to the regular yogurt, especially between 14 and 21 days of storage. Additionally, 7.5% w/v chia mucilage resulted in the highest G′ and G″, indicating increased firmness due to the development of a network from electrostatic interaction between the negative charges of chia mucilage and the positive charges of casein micelles. However, the color and granularity of the yogurts were affected by chia mucilage addition, which was not accepted by the panelists (Ribes et al. 2021). Soybean oil replacement with chia mucilage in mayonnaise was conducted at four concentrations of 15%, 25%, 35%, and 45% by Fernandes and Mellado. In their study, the optimum condition was determined to be 25%, as exceeding this level negatively affected the sensory attributes (Fernandes and Mellado 2018).

Whey protein is of interest in the food industry due to its functional properties. Owing to its amphiphilic nature, it has been introduced as an emulsifier in food systems. Indeed, it interacts with both polar and non‐polar agents in the matrix. This feature is facilitated by applying a shear force to the mixture to help unfold the protein structure and expose the hydrophobic moieties to the environment (Banes et al. 2014). Therefore, it is a candidate in the development of egg‐free and oil‐reduced food products. In this regard, Liu et al. formulated low‐fat mayonnaise with pectin and whey protein isolate, achieving a 40% fat reduction in the formula. They found that the sample containing pectin microparticulate and whey protein isolate had the highest protein content but the lowest sensory score compared to the formula prepared with pectin microparticulate, pectin gel, and full‐fat mayonnaise (Liu et al. 2007).

The development of reduced‐fat and cholesterol‐free (CF) sauces has been achieved using several functional ingredients. However, most of the trials have resulted in impaired sensory attributes, especially in texture and color. To achieve appropriate textural and sensory properties, we needed a water binding network able to entrap water molecules to provide a viscose matrix. Our investigation revealed that no study has explored the use of chia seed mucilage and whey protein in the formulation of egg‐free and reduced‐fat mayonnaise. Chia mucilage was selected to provide a negative charge to interact with the positively charged whey protein under the low pH of mayonnaise. In addition to the development of a three‐dimensional network, whey protein shows emulsification activity to avoid phase separation in the oil‐in‐water formula without egg yolk. Therefore, this study aimed to develop a new formula containing chia seed mucilage and whey protein as alternatives to soybean oil and egg yolk in mayonnaise. In this work, proximate composition, antioxidant activity, rheological properties, and organoleptic attributes were evaluated in the samples.

## 2 Materials and Methods

### 2.1 Chemicals and Culture Media

DPPH solution, Folin–Ciocalteu reagent, calcium carbonate, methanol, hexane, sulfuric acid, hydrochloric acid, boric acid, potassium iodide, acetic acid, chloroform, and sodium thiosulfate were purchased from Biochem company (France). The culture media of Peptone Water, Potato Dextrose Agar, and Plate Count Agar were purchased from Quelab company (Canada). Ingredients of mayonnaise including salt, sugar, mustard powder, soybean oil, vinegar, chia seed, whey protein, and egg yolk were purchased from local markets.

### 2.2 Chia Mucilage Extraction

Chia seeds were added to distilled water in a ratio of 1:20 and left for 30 min at 25°C (ambient temperature). Then, the mixture was filtered by a sieve (mesh no. 18), and the filtrate was centrifuged (Hettich Universal 320, Hettich GmbH, Germany) at 2600 rpm for 20 min. After centrifugation, the supernatant was spread in a flat dish and dried in an oven (Memmert, Germany) at 50°C for 24 h. The dried extract was powdered and stored in a polyethylene bag under cold and dry conditions until analysis (Chaves et al. 2018).

### 2.3 Mayonnaise Production

For the regular mayonnaise, water, salt, sugar, mustard powder, and egg yolk were mixed and stirred for 5 min, followed by the gradual addition of soybean oil. Stirring continued for an additional 10 min. Then, vinegar was added gradually to the mixture, and the final mixture was homogenized with an ultra‐homogenizer (Ultra‐Turrax T25, IKA, Germany) at 12000 rpm for 2 min at ambient temperature (Taslikh et al. 2022). CF mayonnaise was prepared by mixing a portion of water and vinegar. The remaining water was used for the hydration of whey protein. Then, salt, sugar, mustard powder, and whey protein solution were added to the water/vinegar solution and mixed vigorously. After complete mixing, the soybean oil was added, followed by a gradual addition of the remaining vinegar. The final mixture was homogenized with an ultra‐homogenizer for 2 min. The reduced‐fat mayonnaise was prepared by mixing a portion of water, vinegar, salt, sugar, mustard powder, and egg yolk, followed by a gradual addition of soybean oil. The remaining water was used for the hydration of chia mucilage. In the final step, the hydrated mucilage was added to the mixture. For the cholesterol‐free and reduced‐fat (CFRF) mayonnaise, the whey protein solution was prepared with a portion of water and vinegar. Additionally, mucilage powder was dissolved in adequate water. Then, the whey protein solution, salt, sugar, mustard powder, and the remaining water and vinegar were mixed. The soybean oil was added gradually to the mixture, followed by the addition of the hydrated mucilage. The final mixture was homogenized with an ultra‐homogenizer for 2 min. The prepared samples were kept in a refrigerator for 24 h before the experiments. The composition of the samples is presented in Table 1.

**TABLE 1:** Formulation of control, cholesterol‐free, and reduced fat mayonnaise samples.

Ingredient (%w/w)	Control	CF	RF1	RF2	CFRF1	CFRF2
Soybean oil	70	70	50	50	50	50
Egg yolk	10	0	10	10	0	0
Whey protein solution	0	10	0	0	5	5
Chia seed mucilage	0	0	0.5	1	0.5	1
Vinegar	6	6	6	6	6	6
Salt	1.5	1.5	1.5	1.5	1.5	1.5
Sugar	2	2	2	2	2	2
Mustard powder	0.5	0.5	0.5	0.5	0.5	0.5
Water	10	10	29.5	29	34.5	34
Total	100	100	100	100	100	100

Abbreviations: CF, cholesterol‐free sample; CFRF1, cholesterol‐free and reduced fat sample containing 5% whey protein and 0.5% chia mucilage; CFRF2, cholesterol‐free and reduced fat sample containing 5% whey protein and 1% chia mucilage; RF1, reduced‐fat sample containing 0.5% chia mucilage without whey protein; RF2, reduced‐fat sample containing 1% chia mucilage without whey protein.

### 2.4 Proximate Composition Analysis

Fat, protein, moisture, ash, and pH of the samples were determined according to the AOCS methods (American Oil's Chemists Society 1998). Concentration of carbohydrate was determined by subtraction of protein + fat + moisture + ash from 100%.

### 2.5 Emulsion Stability

This experiment was done according to the method of Sinsuwan with some modifications (Sinsuwan 2024). First, 10 g of the sample was shaken at 80°C for 30 min. Then, the mixture was centrifuged at 4000 rpm for 20 min. After centrifuging, the supernatant was removed, and the weight of the precipitate was recorded. Emulsion stability was determined according to Equation (1) as follows:

(1) $$\text{Emulsion stability}\;\left(\%\right)=100–\left[\left(\text{Weight of the supernatant after centrifuge}/\text{weight of the initial sample}\right)\times 100\right]$$

### 2.6 DPPH Assay

DPPH free radical scavenging activity of the chia seed mucilage was determined by mixing 1 mL of DPPH methanolic solution (2.49 mg DPPH in 100 mL methanol 80%) with 50 μL of the extract at different concentrations in the range of 0.5–2.5 mg/mL. The mixture was left in darkness at room temperature for 30 min. Then, the absorbance of the sample and blank (containing methanol instead of the sample in the mixture) was read at 515 nm by spectrophotometer (Unico, UV–Vis 2100, Spain) (Demirkol and Tarakci 2018). The antioxidant activity was determined according to Equation (2).

(2) $$\text{DPPH scavenging}\;\left(\%\right)=\left[\left(\text{Absorbance of blank}–\text{Absorbance of sample}\right)/\text{absorbance of blank}\right]\times 100$$

DPPH inhibition (%) was plotted against the various concentrations of chia seed mucilage, in which the concentration inhibiting 50% of DPPH free radicals was considered as IC_50_.

### 2.7 Total Phenol Content

This experiment was done by mixing 20 μL chia mucilage ethanolic solution (5 g mucilage in 100 mL ethanol 70%), 1580 μL distilled water, and 100 μL Folin–Ciocalteu reagent 2 N. Then, 300 μL of sodium carbonate 7.5% was added, and the mixture was kept in darkness for 2 h. The blank was prepared by methanol, Folin–Ciocalteu reagent, and sodium carbonate. Absorbance of the mixtures was read at 760 nm by spectrophotometer (Unico, UV–Vis 2100, Spain). Gallic acid solution was used for preparation of the calibration curve. Total phenol content was calculated based on mg of gallic acid equivalent per g of chia mucilage according to the following equation (Demirkol and Tarakci 2018; Scapin et al. 2016).

(3) $$\mathrm{TPC}\;\left(\mathrm{mg}/g\right)=c_{1}\times v/m$$

where c_1_ is the concentration of gallic acid (mg/ml) achieved from the calibration curve; v is the volume of the chia mucilage solution (mL); m is the weight of chia mucilage (g).

### 2.8 Peroxide Value

Peroxide value determines the amount of primary oxidation products. It was determined by a titration method as described by Savani et al. (Savani et al. 2023) with minor modification. First, 5 g of the mayonnaise sample was added to a beaker, to which 30 mL of glacial acetic acid and chloroform (3:2) and 0.5 mL of saturated potassium iodide were added. The mixture was left in darkness for 1 min. Then, 30 mL of distilled water and 0.5 mL of starch solution (1% w/v) were added. Titration was done by sodium thiosulfate 0.01 N until discoloration. Peroxide value was determined according to the following formula.

(4) $$\text{Peroxide value}\;\left(\mathrm{meq}\;O_{2}/\mathrm{kg}\;\text{of oil}\right)=\left[\left(V_{2}-V_{1}\right)\times N\times 1000\right]/m$$

where V_1_ and V_2_ are volumes of sodium thiosulfate used in the titration of the blank and sample, respectively; N is the normality of sodium thiosulfate; m is the weight of the sample.

### 2.9 Texture Analysis

Firmness, cohesiveness, consistency, and viscosity were determined by a texture analyzer TA‐XT2 (Surrey, UK) according to the method of Liu et al. (2007). The samples were poured into a 150‐mL graduated cylinder (60 mm internal diameter and 80 mm height), and compressed with a 35‐mm diameter disk. The probe was inserted into the samples up to 2 mm depth at a rate of 1 mm/s, and the force was recorded.

### 2.10 Colorimetry

A Hunter Lab CR 400 colorimeter determined the color of samples based on three parameters of L* for lightness to darkness, a* for red to green, b* for yellow to blue (Savani et al. 2023).

### 2.11 Sensory Evaluation

This test was performed as a preliminary screening by 20 semi‐trained assessors, which is a common practice in pilot food product development studies. A 5‐point acceptability scale was selected in the hedonic method to capture directional differences among treatments rather than to generate full consumer preference data. The assessors evaluated the samples based on color, flavor, and texture. The evaluation was done under sunlight in separate chambers by using water between the tests. The samples were given to the panelists by random codes. They were asked to score the samples based on 1 (extreme dislike), 2 (dislike), 3 (neither like nor dislike), 4 (like), and 5 (extreme like) (Mohammadi et al. 2024). Panelists were provided with the necessary details to understand the study's scope before participating. This guaranteed the study's safety and affirmed that panelists were not subjected to coercion, intimidation, or undue influence. Panelists explicitly agreed prior to the evaluation.

### 2.12 Microbial Analysis

The mayonnaise dilutions of 10^−1^ (10 g of mayonnaise in 90 mL of saline solution 0.85% w/v) and 10^−2^ were prepared in the laboratory. For bacterial test, 1 mL of the dilutions was inoculated in the plate count agar media by pour plating method, and the incubation was done at 30°C for 3 days. Yeasts and molds were enumerated in potato dextrose agar by spreading 0.1 mL of the prepared dilutions on the medium. The medium contained 100 mg/L chloramphenicol to help selective cultivation of fungi. Fungal incubation was done at 25°C for 5 days. The plates containing separate colonies were selected for enumeration. Microbial analysis was done in triplicate. The microbial counts are expressed as log colony forming unit per gram (log CFU/g) of mayonnaise (Hakimian et al. 2022).

### 2.13 Statistical Analysis

The data were analyzed by SPSS software version 19. The means were compared by one‐way ANOVA followed by Duncan post hoc test. Differences were significant at *p* ≤ 0.05. Chemical experiments were done in three replications.

## 3 Results and Discussion

The extract of chia seed mucilage was subjected to antioxidant analysis. Accordingly, IC_50_ of DPPH assay and total phenol content of the chia mucilage were calculated as 1.86 ± 0.5 mg/mL and 4.21 mg GAE/g, respectively. The antioxidant activity of chia mucilage was studied by Atik et al. by adding chia seed mucilage at concentrations of 1%–3% to yogurt, through which they found a significant increase in both DPPH inhibition (2.15%–3.28% vs. 1.91%) and total phenol content (17.03–72.15 vs. 7.02 μg GAE/g) in the presence of chia mucilage compared to the control yogurt (Atik et al. 2020). Comparison of proximate composition between the control and treatment groups showed significant differences in protein, moisture, fat, carbohydrate, and calorie content (Table 2). This was consistent with the findings of Odep et al. in the preparation of reduced‐fat mayonnaise developed with chia mucilage and Arabic gum. The higher carbohydrate content was related to the polysaccharide nature of the mucilage and gum. Moreover, these hydrocolloids bound the water molecules in the environment, increasing the moisture content and viscosity (Odep et al. 2024). The lower calorie content was clearly correlated with the reduced fat level in the samples. Odep et al. found that the pH value of full‐fat mayonnaise was higher than that of reduced‐fat samples, primarily due to the hydrophilic nature of citric acid, which dissociated less in full‐fat mayonnaise. Therefore, the reduced‐fat mayonnaise facilitated citric acid dissociation, leading to a significant pH decrease. However, significant changes in acidity were observed in mayonnaise samples with more than 50% fat. It is assumed that chia mucilage restricts citric acid dissociation by making the water molecules unavailable. It is consistent with the higher viscosity of the reduced‐fat and egg‐free mayonnaise (Table 3). This hypothesis is verified by the lower pH of CF sample developed by whey protein, which provides an environment with the most available water.

**TABLE 2:** Proximate composition (%) and calorie per 100 g of the mayonnaise samples.

Sample	Moisture	Protein	Fat	pH	Carbohydrate	Ash	Calorie
Control	15.21 ± 0.98^a^	2.58 ± 0.23^a^	71.7 ± 1.1^a^	3.92 ± 0.11^a^	9.72 ± 0.24^a^	0.80 ± 0.07^a^	694.41 ± 9.53^a^
CF	19.02 ± 0.87^b^	7.60 ± 0.32^b^	70.8 ± 1.3^a^	3.74 ± 0.13^b^	1.38 ± 0.11^b^	1.20 ± 0.11^b^	673.12 ± 8.76^b^
RF1	30.10 ± 1.07^d^	2.60 ± 0.43^a^	51.1 ± 1.0^b^	3.91 ± 0.10^a^	15.39 ± 0.42^c^	0.81 ± 0.05^a^	532.22 ± 4.65^c^
RF2	28.40 ± 0.97^cd^	2.69 ± 0.21^a^	51.5 ± 0.9^b^	3.91 ± 0.12^a^	16.60 ± 0.54^c^	0.81 ± 0.06^a^	540.66 ± 6.76^c^
CFRF1	29.56 ± 1.02^d^	3.70 ± 0.45^a^	50.7 ± 1.3^b^	3.84 ± 0.11^a^	15.14 ± 0.43^c^	0.90 ± 0.06^c^	531.66 ± 5.76^c^
CFRF2	27.95 ± 0.88^c^	3.69 ± 0.44^a^	50.5 ± 1.1^b^	3.84 ± 0.15^a^	16.95 ± 0.38^c^	0.91 ± 0.07^c^	537.06 ± 5.98^c^

*Note:* Different superscript letters within each column indicate significant differences among samples at *p* < 0.05.

Abbreviations: CF, cholesterol‐free sample; CFRF1, cholesterol‐free and reduced fat sample containing 5% whey protein and 0.5% chia mucilage; CFRF2, cholesterol‐free and reduced fat sample containing 5% whey protein and 1% chia mucilage; RF1, reduced‐fat sample containing 0.5% chia mucilage without whey protein; RF2, reduced‐fat sample containing 1% chia mucilage without whey protein.

**TABLE 3:** Rheology parameters and emulsion stability of the mayonnaise samples.

Sample	Firmness	Consistency	Adhesiveness (mJ)	Viscosity (cp)	Emulsion stability (%)
Control	110 ± 7.5^a^	0.65 ± 0.04^a^	1850 ± 110^a^	8610 ± 109^a^	57.42 ± 0.03^a^
CF	100.2 ± 4.1^a^	0.76 ± 0.08^a^	928 ± 86^b^	4317 ± 63^b^	76.06 ± 0.09^b^
RF1	135.6 ± 10^b^	0.7 ± 0.06^a^	2339 ± 208^cd^	9848 ± 92^c^	96.86 ± 0.04^c^
RF2	135.7 ± 8^b^	0.7 ± 0.05^a^	2565.6 ± 225^c^	10,287 ± 76^d^	97.53 ± 0.05^d^
CFRF1	147.8 ± 6.5^b^	0.73 ± 0.05^a^	1991 ± 140^ad^	8185 ± 42^e^	98.91 ± 0.07^e^
CFRF2	198.2 ± 5.4^c^	0.73 ± 0.04^a^	2170 ± 185^cd^	9657 ± 30^f^	99.08 ± 0.09^f^

*Note:* Different superscript letters within each column indicate significant differences among samples at *p* < 0.05.

Abbreviations: CF, cholesterol‐free sample; CFRF1, cholesterol‐free and reduced fat sample containing 5% whey protein and 0.5% chia mucilage; CFRF2, cholesterol‐free and reduced fat sample containing 5% whey protein and 1% chia mucilage; RF1, reduced‐fat sample containing 0.5% chia mucilage without whey protein; RF2, reduced‐fat sample containing 1% chia mucilage without whey protein.

Rheological properties are important factors affecting consumer acceptance. According to Table 3, except for the CF sample, which showed undesirable rheological parameters, the other treatments developed better texture compared to the control. This demonstrates the importance of chia seed mucilage alone and in combination with whey protein to improve the matrix. Chia seed mucilage is an anionic polysaccharide composed of negatively charged carboxyl groups. These functional groups can interact with positively charged agents in the matrix, allowing the mucilage to act as a stabilizer by increasing the viscosity (Cornejo‐Garcia et al. 2026). In comparison, whey protein can act as an emulsifier to prevent phase separation but is not effective in viscosifying the matrix. Indeed, egg yolk is added to mayonnaise as a gelling and whipping agent and emulsifier. It prevents flocculation and provides an appropriate texture. The strong emulsifying property of egg yolk is driven by low‐density lipoprotein, high‐density lipoprotein, phospholipids, and proteins (Mirzanajafi‐Zanjani et al. 2019; Ma and Boye 2013). Therefore, substituting egg yolk with whey protein cannot improve the textural properties compared to the control mayonnaise due to its insufficient emulsification ability. Hovjecki et al. enriched goat milk yogurt with 1.5% and 3% chia seed mucilage. They found that the higher concentration of chia mucilage increased hysteresis (the energy required for structural disruption). This is likely due to the higher interactions of protein‐polysaccharide in the mixture, which strengthened the matrix. Compared to the control, mucilage addition at lower concentrations may interfere with protein interactions. However, both 1.5% and 3% chia mucilage improved rheological parameters and organoleptic attributes (Hovjecki et al. 2024). As seen in Table 3, the addition of chia mucilage to mayonnaise increased its adhesiveness. However, the best results were achieved by the addition of both chia mucilage and whey protein owing to the protein‐polysaccharide complexation. Under acidic conditions of the matrix (pH < 4), the negatively charged chia mucilage and protonated whey protein formed electrostatic interactions to develop a viscoelastic network (Silva et al. 2022; Ma et al. 2025). Although the functional groups of chia mucilage able to form hydrogen bonds with water molecules have a pivotal role in the development of uniform texture without phase separation (Silva et al. 2022). This aligns with other studies on mayonnaise produced with micro‐fibrillated cellulose, xanthan gum, and modified starch (Blok et al. 2021, 2023). Furthermore, the increased firmness and adhesiveness of the low‐fat and egg‐free mayonnaise agreed with the results of Schadle et al., who added corn dextrin to mayonnaise up to 4% w/w. In their study, the low‐fat mayonnaise samples received significantly higher scores for firmness, stickiness, and creaminess from sensory panelists. Interestingly, these sensory results were strongly supported by the instrumental analysis (Schädle et al. 2022).

The colorimetry assay results revealed that the oil reduction led to decreased yellowness in the products (Table 4). This aligns with the findings of Blok et al. in the preparation of mayonnaise with micro‐fibrillated cellulose, starch, and xanthan gum. In addition, they found that larger oil droplets induced more yellowness in the product (Blok et al. 2023). A similar result was observed by Carcelli et al. when studying reduced‐fat mayonnaise containing a modified corn flour solution (Carcelli et al. 2020). However, all samples remained within the yellow and white range. Moreover, except for the CF mayonnaise formulated with whey protein, which exhibited more redness (Jervis et al. 2012), the other samples tended towards a green color. Similar results were observed by Rojas‐Martin et al. (2023) in the production of low‐fat mayonnaise with *Dioscorea rotundata* hydrocolloid, and by Askari and Mostaghim (2019) in the development of low‐fat mayonnaise with Salep and chitosan. Oil concentration also affects brightness, so that the higher oil results in more brightness in oil‐in‐water emulsion (Abirached et al. 2025). Indeed, the control contained 70% soybean oil, which was emulsified appropriately by 10% egg yolk. In CFRF samples, 20% of oil was removed and the remained oil was emulsified by a portion of whey protein. Therefore, brightness decreased in the treated samples compared to the control, in addition to a higher light scattering in the control compared to the viscous matrix of low‐fat and egg‐free mayonnaise. On the other hand, the complex of protein‐polysaccharide provided large molecules, which scattered less light and resulted in lower brightness (Aksoy Caf and Ersus 2025). In this regard, chia mucilage plays the main role owing to its dual act via electrostatic interaction and hydrogen bond in the mixture. It is in consistence with the results of Table 4, in which the lowest brightness was observed in reduced‐fat mayonnaise containing chia mucilage, followed by CF samples containing whey protein.

**TABLE 4:** The results of instrumental colorimetric assay in the mayonnaise samples.

Sample	L*Black = 0, White = 100	a*Green (−), Red (+)	b*Blue (−), Yellow (+)
Control	79.15 ± 1.03^a^	−1.01 ± 0.5^a^	36.72 ± 1.26^a^
CF	76.55 ± 0.15^b^	1.32 ± 0.08^b^	20.63 ± 0.73^b^
RF1	71.07 ± 1.37^c^	−3.11 ± 0.05^c^	19.71 ± 0.17^c^
RF2	70.55 ± 0.12^c^	−3.15 ± 0.03^c^	19.8 ± 0.08^c^
CFRF1	74.88 ± 0.25^d^	−1.82 ± 0.02^d^	21.43 ± 0.05^d^
CFRF2	74.94 ± 0.12^d^	−1.71 ± 0.01^e^	22.05 ± 0.02^e^

*Note:* Different superscript letters within each column indicate significant differences among samples at *p* < 0.05.

Abbreviations: CF, cholesterol‐free sample; CFRF1, cholesterol‐free and reduced fat sample containing 5% whey protein and 0.5% chia mucilage; CFRF2, cholesterol‐free and reduced fat sample containing 5% whey protein and 1% chia mucilage; RF1, reduced‐fat sample containing 0.5% chia mucilage without whey protein; RF2, reduced‐fat sample containing 1% chia mucilage without whey protein.

The results of the sensory evaluation (Table 5) align with the instrumental analysis. The higher texture scores of the treated samples correspond to the increased viscosity, firmness, and consistency shown in Table 3. Additionally, mouthfeel is directly correlated with the food's texture profile. The improved flavor of egg‐free samples was likely due to the removal of the eggy flavor (Odep et al. 2024). Amin et al. formulated low‐fat mayonnaise with different oil types and gums, where the best sensory evaluation result was related to the use of 0.75%–1% xanthan and guar gum (1:1) and containing 60% sunflower and/or soybean oil. The second‐ranked mayonnaise was the sample containing 45% of each oil, which had a lower odor score than the full‐fat mayonnaise (Amin‐Hala et al. 2014). Except for mouthfeel, the low organoleptic score of the CF sample was consistent with the lowest sensory score of egg‐free mayonnaise formulated with amaranth protein isolate as an egg replacer by Mohammadi et al. (Mohammadi et al. 2024). In their study, the egg‐free sample was rated highest just for mouthfeel. The authors attributed this to its highest viscosity, which was associated with creaminess perception by the panelists. In contrast, the CF mayonnaise had the lowest texture score and viscosity among the samples. It was probably due to the high protein content of amaranth protein isolate containing amino acids able to bond water molecules. Accordingly, appropriate water absorption capacity of amaranth protein isolate was reported in the study of Mohammadi et al., by which could develop a viscose matrix (Mohammadi et al. 2024). Moreover, the lower color score of the treated samples compared to the control was likely due to their reduced brightness.

**TABLE 5:** The results of organoleptic attributes of the mayonnaise samples.

Sample	Color	Flavor	Texture
Control	4.45 ± 0.55ᵃ	3.55 ± 0.75ᶜ	3.85 ± 0.70ᶜ
CF	3.70 ± 0.65ᶜ	3.35 ± 0.80ᶜ	3.20 ± 0.85ᵈ
RF1	3.95 ± 0.60ᵇ	4.05 ± 0.65ᵇ	4.20 ± 0.60ᵇ
RF2	3.90 ± 0.62ᵇ	4.10 ± 0.60ᵇ	4.35 ± 0.55ᵃᵇ
CFRF1	3.85 ± 0.58ᵇᶜ	4.35 ± 0.55ᵃ	4.45 ± 0.50ᵃ
CFRF2	3.80 ± 0.60ᵇᶜ	4.40 ± 0.50ᵃ	4.55 ± 0.45ᵃ

*Note:* Values are expressed as mean ± SD (*n* = 20). Sensory attributes were evaluated using a 5‐point hedonic scale (1 = extreme dislike, 5 = extreme like). Different superscript letters within each column indicate significant differences among samples at *p* < 0.05.

Abbreviations: CF, cholesterol‐free sample; CFRF1, cholesterol‐free and reduced fat sample containing 5% whey protein and 0.5% chia mucilage; CFRF2, cholesterol‐free and reduced fat sample containing 5% whey protein and 1% chia mucilage; RF1, reduced‐fat sample containing 0.5% chia mucilage without whey protein; RF2, reduced‐fat sample containing 1% chia mucilage without whey protein.

Development of the oxidation process is an important quality factor determining the shelf life of mayonnaise (Merkx et al. 2021; Wills and Cheong 1979). Overall consideration of the samples revealed that those formulated with both whey protein and chia mucilage were superior to the others. Therefore, they were selected for further investigations during storage. Despite the similar peroxide value of the control and treated mayonnaise samples on day 1, the treated samples showed decreased oxidation during 60 days of storage, resulting in greater differences over time. It was related to the lower unsaturated fat levels susceptible to oxidation in the treated samples. In addition, the antioxidant potency of chia seed mucilage cannot be ignored. As seen in Table 6, the sample containing 1% chia mucilage (CFRF2) consistently had a lower peroxide value than CFRF1 containing 0.5% chia mucilage. This is of industrial importance in developing light mayonnaise, where extended shelf life could be achieved with improved value‐added outcomes. Moreover, the product can be used by a wider range of consumers suffering from high blood lipid levels, overweight, and obesity, with less concern about inflammatory symptoms after intake. Roshandel et al. formulated low‐fat mayonnaise with oleaster flour at levels of 4%, 6%, and 8%, achieving a 10%–40% fat reduction. Similar to our results, a lower peroxide value was observed in their low‐fat mayonnaise compared to the control prepared with TBHQ as an industrial synthetic antioxidant. The authors believed that the viscous matrix developed by the fat‐replacer, which favored lower oxygen diffusion into the matrix, was associated with the lower peroxide value (Roshandel et al. 2023). Moreover, phenolic compounds containing hydroxyl groups, responsible for free radical scavenging (Roshandel et al. 2023; Gorji et al. 2016), were found in oleaster flour in the study by Roshandel et al. (2023).

**TABLE 6:** Peroxide value (meq O_2_/kg of oil) of the mayonnaise samples during 60 days of storage.

Sample	Day 1	Day 15	Day 30	Day 45	Day 60
Control	2.73 ± 0.03^a^	3.15 ± 0.05^a^	4.61 ± 0.06^a^	5.29 ± 0.06^a^	5.86 ± 0.06^a^
CFRF1	2.73 ± 0.03^a^	2.92 ± 0.04^b^	3.25 ± 0.05^b^	3.55 ± 0.05^b^	3.74 ± 0.05^b^
CFRF2	2.73 ± 0.03^a^	2.85 ± 0.04^b^	3.10 ± 0.04^b^	3.26 ± 0.05^b^	3.30 ± 0.05^b^

*Note:* Different superscript letters within each column indicate significant differences among samples at *p* < 0.05.

Abbreviations: CFRF1, cholesterol‐free and reduced fat sample containing 5% whey protein and 0.5% chia mucilage; CFRF2, cholesterol‐free and reduced fat sample containing 5% whey protein and 1% chia mucilage.

Mayonnaise has a pH lower than 4, which provides an acidic environment that limits bacterial growth. However, fungal growth must be restricted by using qualified raw materials and good manufacturing processes. According to Table 7, bacterial growth remained well below the maximum permitted level of 3 log CFU/g during 28 days of storage. In comparison, mold and yeast growth was consistently lower than the maximum permitted levels of 2 log CFU/g. A comparison of full‐fat and treated mayonnaise samples showed a negligible increase in growth in the light mayonnaise samples. Similar results were observed in low‐fat mayonnaise formulated with potato powder solution (El‐Bostany et al. 2011). This was due to the lower water content or moisture in the control (Table 2), which limited microbial growth (Roller and Covill 2000). However, it is assumed that the higher water content in the other samples was bound by the mucilage and protein network. Accordingly, most of the water molecules are likely unavailable to microorganisms (Ma and Boye 2013). Thus, no significant difference in microbial growth is seen between the light and full‐fat mayonnaise samples.

**TABLE 7:** The results of microbial analysis (log CFU/g) of the mayonnaise samples during 28 days of storage.

Sample	Day	Total count	Molds and yeasts
Control	1	0.23 ± 0.015^a^	1.22 ± 0.015^a^
	14	0.28 ± 0.022^a^	1.27 ± 0.020^a^
	28	0.33 ± 0.034^a^	1.38 ± 0.035^a^
CFRF1	1	0.23 ± 0.018^a^	1.22 ± 0.017^a^
	14	0.31 ± 0.023^a^	1.33 ± 0.025^a^
	28	0.39 ± 0.032^a^	1.47 ± 0.034^a^
CFRF2	1	0.23 ± 0.020^a^	1.22 ± 0.016^a^
	14	0.29 ± 0.021^a^	1.29 ± 0.022^a^
	28	0.36 ± 0.030^a^	1.40 ± 0.033^a^

*Note:* Same superscript letters within each column indicate significant differences among samples at *p* < 0.05.

Abbreviations: CFRF1, cholesterol‐free and reduced fat sample containing 5% whey protein and 0.5% chia mucilage; CFRF2, cholesterol‐free and reduced fat sample containing 5% whey protein and 1% chia mucilage.

## 4 Conclusion

Egg yolk removal and partial substitution of soybean oil (up to 20%) with chia seed mucilage (0.5%–1%) and whey protein (5%–10%) in mayonnaise yielded promising results in our study. Our findings revealed that the new formula had a lower peroxide value than the control, mainly due to the phenol content and free radical scavenging activity of chia seed mucilage. Additionally, better emulsion stability was observed in the samples formulated with chia seed mucilage and whey protein without egg yolk and containing a reduced level of soybean oil. The more appropriate textural properties in the light mayonnaise samples were associated with the protein‐polysaccharide complexation driven by electrostatic interactions, development of hydrogen bonds between chia mucilage functional groups and water molecules, and emulsifying activity of whey protein in the matrix. On the other hand, fat reduction and egg substitution resulted in equal or better sensory attributes and lower calories in the samples. We concluded that both quality parameters and sensory attributes improved in CFRF mayonnaise. Importantly, the novel formula was cost‐effective with healthier attributes compared to full‐fat mayonnaise, which can be used industrially to help the consumers in preparation of a nutritious food basket. Moreover, egg yolk and vegetable oil are susceptible foods, and their removal/substitution will prolong the shelf life of the product, which facilitates further industrialization and scaling up. Our work includes some limitations. It was a preliminary acceptability and difference‐direction screening study to find the best‐fitted sample compared to the control (full‐fat mayonnaise) with the least calories and health‐threatening side effects. Therefore, a least number of 20 semi‐trained sensory assessors were recruited. Moreover, microstructure studies including droplet size distribution measurements and scanning electron microscopy were not performed in our study. Therefore, interpretation of color changes was limited to the empirical observations. Further research focusing on these limitations and considering more quality factors such as secondary oxidation products is recommended in the future.

## Author Contributions

**Abdollah Yousefi Sabet:** conceptualization, investigation, methodology, formal analysis. **Ashkan Jebelli Javan:** conceptualization, investigation, methodology, visualization, supervision, validation, data curation. **Ali Mahdavi:** supervision, project administration, visualization. **Masoumeh Moslemi:** conceptualization, writing – original draft, validation, writing – review and editing, formal analysis, data curation, investigation.

## Funding

The authors have nothing to report.

## Conflicts of Interest

The authors declare no conflicts of interest.

## Data Availability

The data that support the findings of this study are available from the corresponding author upon reasonable request.
